# Community terminal restriction fragment length polymorphisms reveal insights into the diversity and dynamics of leaf endophytic bacteria

**DOI:** 10.1186/1471-2180-13-1

**Published:** 2013-01-03

**Authors:** Tao Ding, Michael W Palmer, Ulrich Melcher

**Affiliations:** 1 Department of Biochemistry and Molecular Biology, Oklahoma State University, Stillwater, OK, 74078, USA; 2 Department of Botany, Oklahoma State University, Stillwater, OK, 74078, USA

**Keywords:** Leaf bacterial endophytes, Ecology, T-RFLP, Biodiversity

## Abstract

**Background:**

Plant endophytic bacteria play an important role benefiting plant growth or being pathogenic to plants or organisms that consume those plants. Multiple species of bacteria have been found co-inhabiting plants, both cultivated and wild, with viruses and fungi. For these reasons, a general understanding of plant endophytic microbial communities and their diversity is necessary. A key issue is how the distributions of these bacteria vary with location, with plant species, with individual plants and with plant growing season.

**Results:**

Five common plant species were collected monthly for four months in the summer of 2010, with replicates from four different sampling sites in the Tallgrass Prairie Preserve in Osage County, Oklahoma, USA. Metagenomic DNA was extracted from ground, washed plant leaf samples, and fragments of the bacterial 16S rDNA genes were amplified for analysis of terminal restriction fragment length polymorphism (T-RFLP). We performed mono-digestion T-RFLP with restriction endonuclease *Dde*I, to reveal the structures of leaf endophytic bacterial communities, to identify the differences between plant-associated bacterial communities in different plant species or environments, and to explore factors affecting the bacterial distribution. We tested the impacts of three major factors on the leaf endophytic bacterial communities, including host plant species, sampling dates and sampling locations.

**Conclusions:**

Results indicated that all of the three factors were significantly related (α = 0.05) to the distribution of leaf endophytic bacteria, with host species being the most important, followed by sampling dates and sampling locations.

## Background

Bacteria are associated with plants in many ways. They include rhizosphere bacteria that are found in the soil surrounding roots, rhizoplane bacteria that reside on the root surfaces and phyllosphere bacteria that are associated with leaves. Within each of these spheres of plant influence, it is common to distinguish between those bacteria that are associated loosely with the outside of the roots or leaves, the epiphytes, from those that have colonized the internal parts of the organs, the endophytes. Rhizoplane bacteria have been extensively studied, as have root endophytic bacteria [1-3]. Numerous publications address leaf epiphytic bacteria [4-6]. Only few studies have examined specifically leaf endophytic bacteria as part of phyllosphere bacteria [7]. The diversity of leaf endophytic bacteria in different plants is largely unexplored, and is the main subject of this study. We want to understand what factors shape the communities of leaf endophytic bacteria.

A universally accepted definition of plant endophytic bacteria has not been established. In this study, we follow Hallmann’s definition of endophytic bacteria [8] as those bacteria that “can be isolated from surface-disinfested plant tissue or extracted from within the plant and do not visibly harm the plant”. Endophytic bacteria have been found in most plants, colonize the internal tissues and construct diverse relationships with their host plants. Endophytic bacteria can be beneficial to the host plant, including by growth promotion [9], biological control against plant pathogens [8], and bioremediation of the contaminated environment [9]. Although non-pathogenic to host plants, some endophytic bacteria may have the potential to become pathogens [1] to other plants, and may be harmful to animals or even humans. Assessing this potential requires gathering a general understanding of endophytic microbial communities, their diversity, and their distribution among plant species, plant individuals and plant organs.

Traditionally, most studies of endophytic bacterial communities [10-12] are based on bacterial culture methods. However, most environmental bacteria are not cultivable, as evidenced, for example, by the finding that culture-independent methods revealed a broader diversity of bacteria than did culture-dependent methods in a study of bacteria in the apple phyllosphere [13]. In recent years, the study of endophytic bacteria often has employed culture-independent methods, most of which are based on the PCR amplification of bacterial 16S rDNA. Some notable studies of root endophytic bacteria [2,14,15] focused on single crop species, including maize and rice, because of their importance to food supply and safety. Several researchers have applied Terminal Restriction Fragment Length Polymorphism (T-RFLP) [16], a rapid fingerprint technique based on 16S rDNA PCR, to the evaluation of endophytic bacteria. T-RFLP can compare multiple microbial communities fast and accurately, especially when high-throughput bacterial community characterization is needed.

In this project, we studied leaf endophytic bacteria in diverse environments from the Tallgrass Prairie Preserve (TGPP), Osage County, Oklahoma, USA [2], managed by The Nature Conservancy, and which was the site of previous efforts by a Plant Virus Biodiversity and Ecology team to examine the diversity of viruses associated with plants growing in this setting [17]. That study showed nucleotide sequence evidence of bacterial association with plants [17-19]. We extracted total DNAs from plant samples obtained in the TGPP and amplified bacterial 16S rDNA sequences using bacterial rDNA specific primers. Rather than using multi-digestion T-RFLP with three or more restriction endonucleases, we performed mono-digestion T-RFLP with restriction endonuclease *Dde*I, to reveal the structures of leaf endophytic bacterial communities, to identify the differences between plant-associated bacterial communities in different plant species or environments, and to explore the factors affecting the bacterial distribution.

## Methods

### Plant sampling

Healthy, above-ground parts of plant samples were collected monthly from May to August, 2010, in the TGPP). Four sites were randomly chosen (Additional file 1: Table S1). At each site, samples of 5 species of plants (*Asclepias viridis*, *Ambrosia psilostachya*, *Sorghastrum nutans*, *Panicum virgatum*, and *Ruellia humilis*) that are among the most frequent in the TGPP were collected. At each site, three multi-branched individuals of *A*. *viridis* were identified and labeled with tags on May 14^th^ 2010, and one branch was harvested. On June 16^th^ and July 14^th^ (in August *A*.*viridis* samples were not found in the TGPP due to senescence), additional branches were removed for processing. One individual of each of the other four species was collected at each site in four consecutive months from May to August. Healthy leaves were collected and processed for DNA extraction.

### Extraction of total DNA from plants

All leaves were recovered from each plant sample and then washed with running tap water for at least 5 min to remove soil, dust and epiphytic organisms, followed by shaking in 75% ethanol twice each for 3 min, and then rinsed with running distilled water for 3 min. To validate the effect of the protocol, treated leaves were rinsed with 10 ml double distilled water for 3 min. The rinse water was collected and incubated on Lysogeny Broth (LB) plates at 37% overnight. No colonies were observed. Treated leaf samples were ground into a fine powder with liquid nitrogen. Then, 0.1 g of the grindate was resuspended in a 1.5 ml microcentrifuge tube containing 1 ml CTAB extraction buffer [2% (w/v) cetyltrimethylammonium bromide, CTAB; 100 mM Tris–HCl (pH 8.0), 1.4 M NaCl, 20 mM EDTA, 1.5% polyvinyl-pyrolidone, PVP; 0.5% 2-mercaptoethanol] preheated to 65%. Contents were mixed by inverting the tube several times, followed by incubating the tubes in a 60% water bath for 60 min. The tube was centrifuged at 12,000 rpm for 5 min at 4°C and the supernatant was transferred to a new tube. DNA was then extracted twice with chloroform-isoamylalcohol (24:1 v/v) until the aqueous phase was clear. DNA was precipitated using 2 to 2.5 volumes of absolute ethanol, and 0.1 volume 3 M sodium acetate for 2 h at −20°C, followed by centrifugation at 12,000 g for 10 min at 4°C, washed with 1 ml DNA wash solution (0.1 M trisodium citrate in 10% ethanol) twice (30 min incubation and 5 min centrifugation) and 1.5 ml 75% ethanol once (15 min incubation and 5 min centrifugation), then air dried. Finally, DNA was resuspended in 50 μl DNase-free water.

### PCR amplification

Because the bacterial 16S rDNA sequences are highly similar to plant mitochondrial and chloroplast rDNA sequences, popular universal bacterial 16S rDNA primers are not appropriate for specific amplification of bacterial rDNA from plant DNA extracts [20]. Primers 799F and 1492R [14] designed to exclude amplification of plastid 16S rDNA, were used in PCR. Each 50 μl PCR contained PCR buffer (Promega, MadisonWI), 2.5 mM MgCl2, 200 μM each dNTP, 0.5 mg/ml BSA, 15 pmol of each primer, and 2.5 U Taq polymerase. Thermal cycling conditions were: an initial denaturation at 95°C for 3 min followed by 30 cycles of 94°C for 20 sec, 53°C for 40 sec, 72°C for 40 sec, and a final extension at 72°C for 7 min. The PCR yielded a 1.1 kbp mitochondrial product and a 0.74 kbp bacterial product. These were electrophoretically separated in an agarose gel and recovered from the gel using Qiaquick gel extraction kit (Qiagen). Bacterial rDNA amplicons from multiple PCRs from the same template were pooled for restriction.

### The selection of restriction endonuclease and T-RFLP

Engebretson et al. [21] suggested that four restriction endonucleases including *Bst*UI, *Dde*I, *Sau*96I, and *Msp*I had the highest frequency of resolving single populations from bacterial communities. To select the endonuclease with the highest power to resolve leaf endophytic bacterial communities, we cloned 16 s rDNA PCR products and randomly selected and sequenced inserts from 50 colonies. Computer-simulated virtual digestions indicated that *Dde*I generated the most distinct T-RFs and thus had the highest resolution. Therefore, we chose *Dde*I (Promega) to perform the mono-digestion T-RFLP to generate T-RFLP profiles from five species of plants.

Restriction digestion reactions were incubated at 37°C for 4 h, followed by 20 min at 65°C to denature the enzyme. Two microliters of the restricted PCR product were mixed with 0.75 μl of size standard LIZ1200 (ABI, Foster City, CA) and 7.25 μl of Hi-Di formamide (ABI). DNA fragments were scanned on an ABI 3730 automated DNA sequencer at Oklahoma State University’s Recombinant DNA/Protein Core Facility. The T-RFLP data profiles were obtained and analyzed by using GeneMapper Software version 4.0 (ABI).

### Data processing and statistical analysis

In 16S-rDNA-T-RFLP profiles, a baseline threshold of 50 relative fluorescence units was used to distinguish ‘true peaks’ from background noise. Considering T-RF drift (improperly sized T-RFs due to differences in fragment migration and purine content), peaks were manually aligned using the method described by Culman et al. [22]. After background removal, raw peak height was normalized to balance the uncontrolled differences in the amount of DNA between samples by dividing the peak height by the sum of all peak heights of each sample. Culman et al. [22] determined that relative peak heights are better than peak areas for comparisons in T-RFLP profile analysis, yielding greater signal to noise ratios.

All the T-RFLP data were arranged into a matrix with each row as a community sample and each column as the relative abundance of each T-RF. The matrix was analyzed by partial Canonical Correspondence Analyses (pCCA) using *Canoco for Windows* 4.5 (Plant Research International) (32). We performed three kinds of pCCAs: using, as explanatory variables: sites, months, and host species. For each of these analyses, the other variables (e.g. for the third analysis, months and sites) were used as covariables. This approach allowed us to isolate the independent effects of each factor. For each analysis, we performed a permutation test of significance with 9,999 permutations, conditioned on the covariables.

Based on the complete T-RFLP data matrix, we calculated also the percentage of empty cells in the data matrix [23] as 100% x (total number of cells in the data matrix of T-RFs vs. samples - count of all cells with non-zero values)/(total number of cells in data matrix). Multivariate Analysis of Variance (MANOVA) was conducted using SAS v9.2 (SAS Institute Inc.) and Hierarchical Clustering Analysis was carried out with R (R development core team, 2003).

The average proportion per existence (APE) of all T-RFs found in five host species estimated the prevalence of T-RFs in diverse communities. APE is defined as the average proportion of one T-RF over those host samples which contain this T-RF in their T-RFLP profiles, and was calculated by the sum of the relative proportions divided by the number of the samples containing this T-RF, as in the following formula:

$$\mathrm{APE}=\frac{\sum_{i=1}^{m}\mathrm{Pi}}{n}$$

where *Pi* is the relative proportion of the T-RF in *i*th sample, *m* is the total number of samples, and *n* is the number of these which have the T-RF.

## Results

### Mono-digestion T-RFLP

In this study, we used T-RFLP profiles to study the features of the distribution of leaf endophytic bacterial communities. Rather than using multiple restriction digestions and then comparing the combined T-RFLP profiles to entries in a pre-computed database, here we chose to use only one restriction endonuclease and the T-RFs with a certain length were treated as a special kind of OTU (Operational Taxonomic Unit) - Operational T-RFLP Unit, a unit that can be directly used to describe a community. In this manner we avoided the problems caused by T-RFs not referring to a known bacterial species in the database. This approach allows direct study of the complexity of, and changes in, distribution of leaf endophytic bacteria without requiring taxonomic identification.

Osborn et al. [24] have demonstrated that T-RFLP is highly reproducible and robust in studying microbial communities and yields high-quality fingerprints consisting of fragments of precise sizes. In this research we also confirmed the reproducibility of T-RFLP to validate the application of T-RFLP to study endophytic bacterial communities. We repeated the complete procedure from DNA extraction to final T-RFLP scanning, and the results indicated that the T-RFLP profiles from the same sample were indistinguishable (Additional file 2: Figure S1).

### General analysis of T-RFLP profiles of endophytic bacterial communities in *A*. *viridis*

We focus first on *A*. *viridis* for two reasons. The anatomy of the plant allowed us to resample the same individual over three months. Further, this species is a major host of Asclepias asymptomatic virus, one of the most prevalent viruses of the TGPP [25] and one that may impact endophyte compositions. In total, we obtained 36 *A*. *viridis* samples from four sites, sampled monthly from May to July with three samples for each site. T-RFLP profiles were generated for all and analyzed to identify T-RFs. The analysis of those T-RFLP profiles enabled us to determine the effect of sampling date and sites on the composition of endophytic bacterial communities within one host plant species. The total number of T-RFs increased from May to July, suggesting that as the plant grows from May to July, endophytic bacteria become more diverse (Table 1). The richness of T-RFs (defined as the average number of T-RFs in a dataset) of samples from May, much lower than of those from June and July, indicated that from May to June, the complexity of the endophytic bacterial community increased three-fold. The percentage of empty cells [23] is a measure of sharing of community components [21]. Samples from May had the highest percentage, while samples from June had the lowest percentage, suggesting that in June different host plants share more common leaf endophytic bacterial species than they do in May, consistent with the leaf endophytic bacterial communities in June being more complex.

**Table 1:** **Summary statistics for T-RFs of *Asclepias viridis *samples from different months and sites**

**Sample variable^a^**	**Total T-RFs**	**Richness**	**Percent empty cells in matrix**	**Beta diversity**
Data summarized by months				
May	27	6.8	77.2%	2.95
June	46	21.9	52.3%	1.10
July	59	20.0	68.7%	1.95
Data summarized by sites				
Site 1	45	15.3	65.9%	1.93
Site 2	44	15.4	64.9%	1.76
Site 3	44	15.0	65.9%	1.93
Site 4	33	13.8	58.2%	1.39

^a^For months, data summarized over all sites; for sites, data summarized over all months.

**Figure 1:**
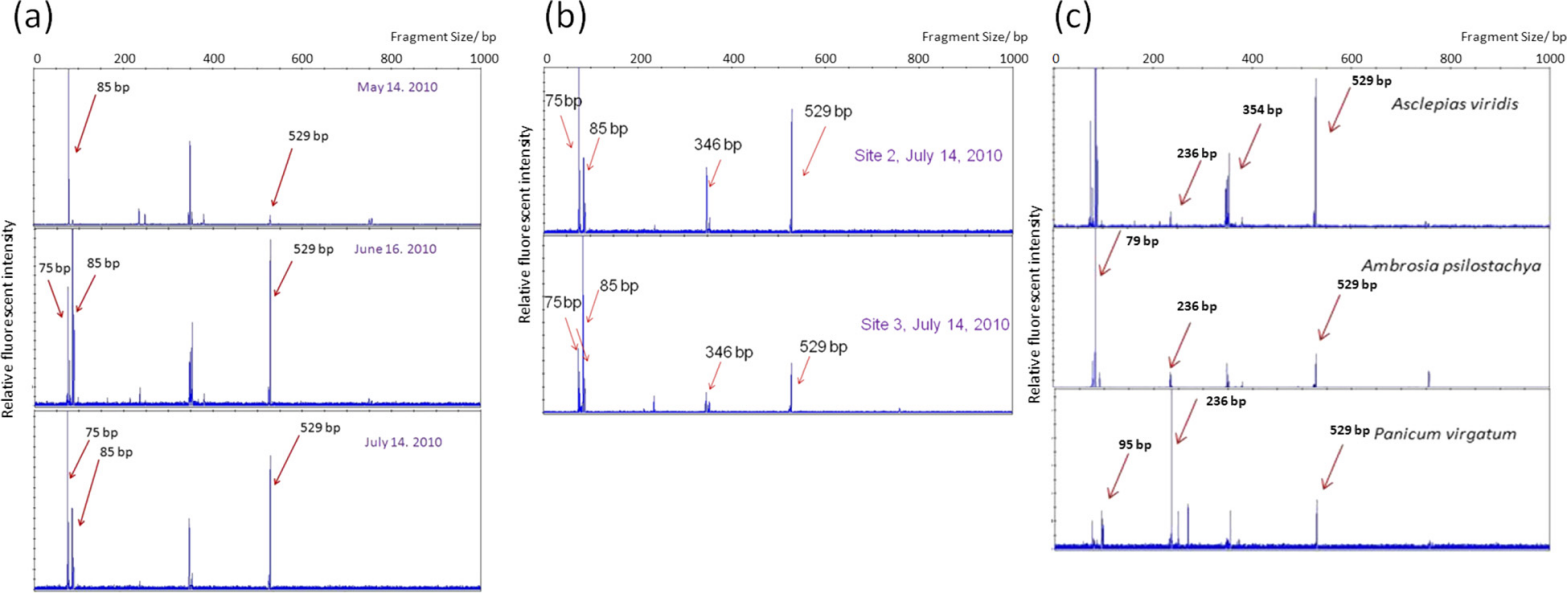
**Comparisons of T-RFLP profiles of endophytic bacterial communities.** Relative fluorescence intensity (normalized to the most intense peak) is plotted against length of the T-RF. T-RFLP profiles represented the bacterial species compositions, indicating the influences from multiple factors: (**a**) T-RFLP profiles from one tagged *A*. *viridis* individual, samples of which were collected respectively on May 14th, June 16th and July 14th, 2010. (**b**) T-RFLP profiles from two *A*. *viridis* individuals respectively from Site 2 and Site 3, both collected on July 14th, 2010. (**c**) Selected T-RFLP profiles from 3 individuals respectively from *A*. *viridis*, *A*. *psilostachya* and *P*. *virgatum*. For the dominant T-RFs from these three plant species, see Additional file 1: Table S2.

Temporal variations of leaf endophytic bacteria were also observed in T-RFLP patterns, which reveal the development of different T-RFs during the growing season. We labeled three *A*. *viridis* plants at each site in order to track the dynamics of the leaf endophytic bacterial community of the same host plants. Figure 1(a) shows the comparison of T-RFLP patterns of one *A*. *viridis* individual from May to July. On May 14, the dominant T-RF in this bacterial community was the T-RF 85 bp. On June 16, an increase of the relative abundance of the T-RF 529 bp led this T-RF to share dominance of this bacterial community with the T-RF 85 bp. On July 14^th^, the dominance of the T-RF 85 bp had been replaced by the T-RF 75 bp, which had a significant increase in relative abundance from May to July. The observations indicate that the leaf endophytic bacterial community changed with the season.

### *A*. *viridis* T-RFLP pattern variation contributed by sampling sites and dates

Unlike the samples from different months, the samples from different sites did not show significant variation when the data were analyzed for the presence or absence of individual T-RFs (Table 1) even though samples from site 4 appeared to have a lower diversity of leaf endophytic bacteria than others. Although the general level of diversity of leaf endophytic bacteria did not show variation among sites when presence/absence data were considered, the T-RFLP profiles of samples from different sites suggested that the compositions and the relative abundances of individual T-RFs varied with the site/location of host plants, revealing a possible connection of leaf endophytic bacterial species with host locations. Figure 1(b) shows the T-RFLP patterns of two *A*. *viridis* plants both collected on July 14, 2010, but from different sites. In the sample from site 2, the T-RF 75 bp was more prominent than the T-RF 85 bp; while in the sample from site 3, the T-RF 85 bp was more prominent. Other dominant T-RFs, including the T-RF 364 bp and the T-RF 529 bp, also show differences in relative abundance. The influence of host locations may contribute to differences in endophytic bacterial community compositions. Alternatively, the differences could reflect sample to sample variation.

### Partial canonical correspondence analysis (pCCA) of T-RFLP profiles

**Figure 2:**
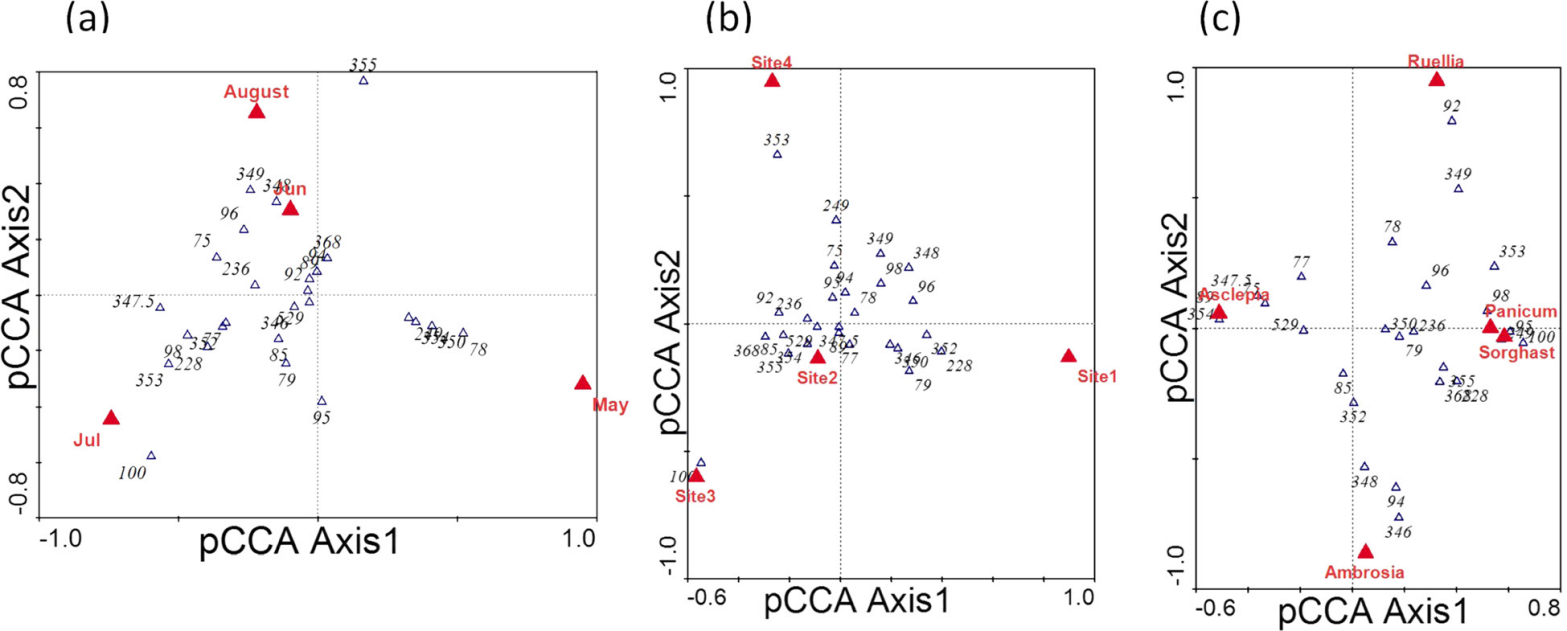
**Partial Canonical Correspondence Analyses (pCCA) of T-RFLP profiles treating each of the three factors considered as the environmental factor. **(**a**) pCCA of T-RFLP profiles of *A*. *viridis* samples treating sampling date as the environmental factor. (**b**) pCCA of T-RFLP profiles of *A*. *viridis* treating sampling location as the environmental factor. (**c**) pCCA of T-RFLP profiles of all five host species samples treating host plant species as the environmental factor. The pCCA indicated that the three factors tested were all significant. pCCA Axes1 and 2 represent the two most important canonical correlations that explain the sample variation with pCCA Axis1 being the most important.

As described above, endophytic bacterial communities varied with the time of sampling and the locations of host plants. To determine the relative importance of each factor, the relative abundances of each T-RF were used to conduct pCCA of T-RFLP profiles. Figure 2 (a) shows the pCCA of T-RFLP profiles of *A*. *viridis* treating sampling dates as the environmental factor with sampling locations as covariable. Because the first pCCA axis is more important than the second axis, the differences between samples from May and the other two months are more significant than the differences between samples from June and July, a result which is consistent with the summary statistics of T-RFs (Table 1). This result implies rapid early changes in the development of endophytic bacterial communities, consistent with rapid plant growth of the host species, *A*. *viridis*. Permutation tests revealed sampling date is a significant factor (p-value = 0.0001).

The pCCA result of T-RFLP profiles of *A*. *viridis* treating location of host plants as environmental factor with sampling dates as covariable (Figure 2 (b)) indicated that the differences between samples from site 1 and other sites were stronger than the differences between sites 2 and 3. Permutation tests revealed location of host plants was a significant factor (p-value = 0.0005).

### Extension of the analysis to multiple host species

Having established month to month variation and sites as significant factors shaping endophytic bacterial communities in *A*. *viridis*, we asked whether the *A*. *viridis* communities were shared in other species growing at the same times in the same locations and whether those species had similar time and location influences on their community compositions. Host plant species may influence leaf endophytic bacterial communities because of their different physiological and biochemical features. Indeed, the T-RFLP patterns of *A*. *viridis*, *A*. *psilostachya*, and *P*. *virgatum* individuals were distinct (Figure 1(c)). The total number of T-RFs detected varied from 16 for *R*. *humilis* to 72 for *A*. *viridis* (Additional file 1: Table S3). The beta-diversity calculated for each host species was significantly lower than the diversity when samples were grouped by sample date or site (Additional file 1: Table S3). The dominant T-RFs (the group of the T-RFs which have an average proportion more than 3% of the total) for these three species (Additional file 1: Table S2) reveal that each host species had its own characteristic group of dominant T-RFs. Especially the most dominant T-RFs differed among these three species. These observations indicate that the host species has properties determining the compositions of their leaf endophytic bacterial populations.

The pCCA result of treating host species as the environmental factor with sampling dates and locations as covariables in analyzing T-RFLP profiles using data from five host plant species supports that T-RF patterns are influenced by the host species identity (Figure 2 (c)). In the pCCA biplots, *S*. *nutans* and *P*. *virgatum* were close to each other, indicating that the leaf endophytic bacterial communities from these two species were similar to each other. Those of the other three host species were distinct from each other with *A*. *viridis* the most distinct, since the data point of *A*. *viridis* lay on the other end of the first axis. The analysis was performed also using only May, June and July data to guard against bias introduced by the absence of *A*. *viridis* August data. The results were essentially the same. These results are consistent with the features of the host plant species: both *S*. *nutans* and *P*. *virgatum* are grass species; *A*. *viridis* is different from the other four species because it contains latex, giving it the common name “milkweed”. Permutation tests revealed host species as a significant factor (*p*-value = 0.0001).

We also studied the impacts of the sampling dates and host plant locations based on the 5-species dataset using pCCA. Results (data not shown) indicate that both of these factors were also significant with *p*-values < 0.01. The 5-species pCCA biplots confirm the inference we obtained from the *A*. *viridis* pCCA biplots, that samples from May were more distinct from other samples considering sampling date as an environmental factor, and samples from Site 1 were more distinct from other samples considering sampling site as an environmental factor. After an analysis using all three factors as environmental factors, we were able also to partition the overall variation to reveal how much variation was contributed by each factor. Results calculated from pCCA eigenvalues indicated that host plant species contributed 49.8% of the overall variation, sampling date contributed 28.5%, and host plant locations contributed 14.2%. Thus although these three factors all significantly determined the structure of endophytic bacteria, host plant species was the most important factor, followed by sampling date and host locations.

### Statistical analyses of the diversity of leaf endophytic bacteria

The diversity of leaf endophytic bacteria was examined also by counting the number of T-RFs in each community. The average number of T-RFs (Table 2) over all samples of *R*. *humilis* was significantly smaller than those of *A*. *psilostachya*, *P*. *virgatum* and *A*. *viridis* by Tukey range test (*p* = 0.0014). This result indicates that *R*. *humilis* plants have a simpler endophytic bacterial community than the other species. This result further supports that the host plant species plays an important role in determining the diversity of endophytic bacteria. The average number of T-RFs (Table 2) appeared to have risen from May to July and then fallen from July to August. However, the Tukey test did not detect any significant differences among these four different months. The Tukey test also did not detect any significant differences among the average number of T-RFs in the four sites (Table 2). However we cannot rule out significant differences had a larger spatial scale been chosen. The tests agree with the pCCA results described above: the host plant species is the most important factor. Considering that average numbers of T-RFs are unweighted alpha diversity indices, the weighted alpha diversity indices (Shannon indices) were also calculated based on the relative proportions of each T-RFs (Additional file 3: Table S4). These indices also supported the conclusion that the host species was the most important factor.

**Table 2:** Average numbers of T-RFs of endophytic bacterial communities from each host plant species, sampling date and location

**Samples**	**Average number of T-RFs**
Data collated by host species	
*Ambrosia psilostachya*	17.38 +/− 4.98
*Panicum virgatum*	15.00 +/− 10.46
*Asclepias viridis*	14.89 +/− 7.04
*Sorghastrum nutans*	12.92 +/− 5.09
*Ruellia humilis*	5.50 +/− 2.72
Data collated by site	
Site 1 Samples	14.71 +/− 7.46
Site 2 Samples	13.86 +/− 6.94
Site 3 Samples	12.45 +/− 7.84
Site 4 Samples	14.60 +/− 8.24
Data collated by sampling date	
May Samples	9.29 +/− 7.95
June Samples	14.72 +/− 6.16
July Samples	18.04 +/− 5.91
August Samples	12.73 +/− 7.47

**Figure 3:**
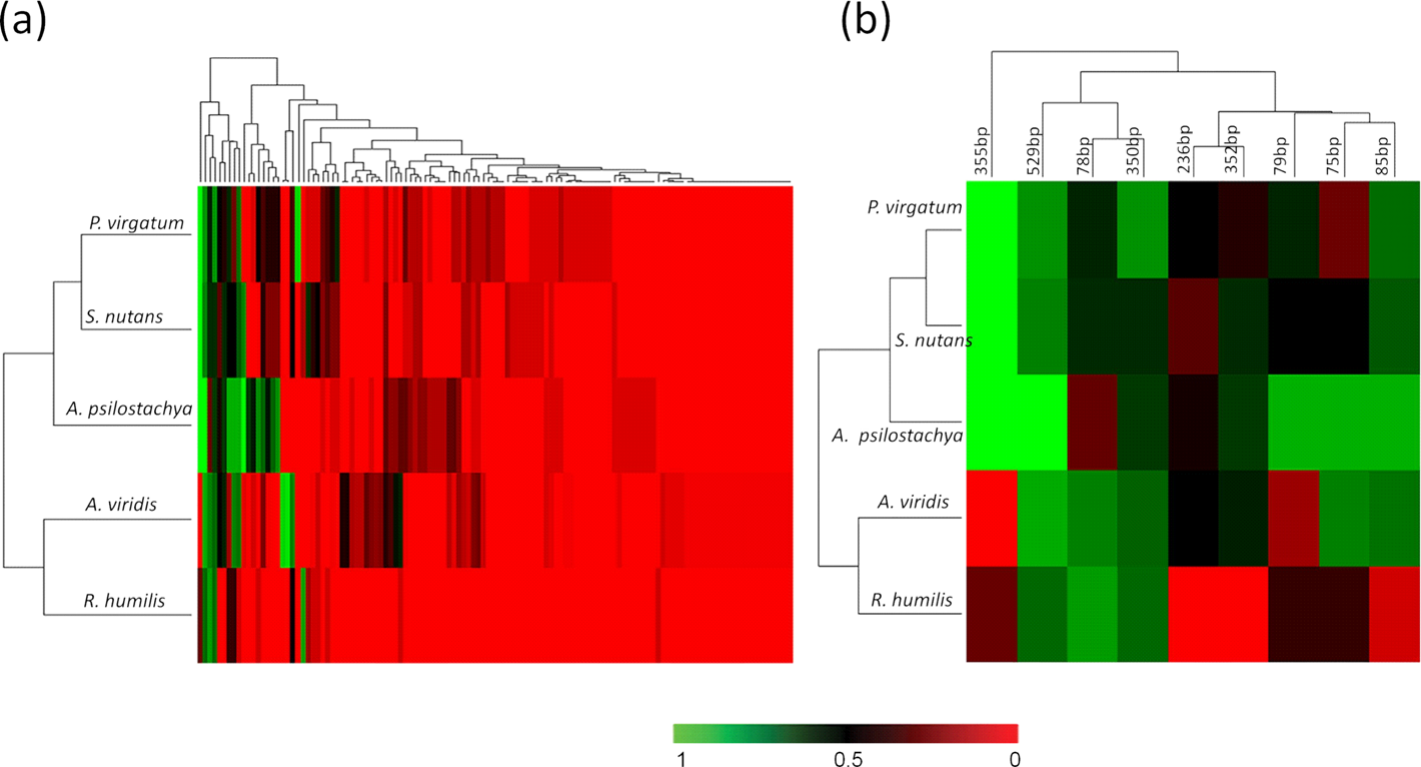
**Heatmap of the frequencies of T-RFs detected in five host species. **(**a**) The complete heatmap showed the frequencies of all the T-RFs and the clustering results of the T-RFs and host species. (**b**) The first branch of the clustering of the T-RFs in (**a**) containing most frequent T-RFs. The color change from green to red indicates the frequency changing from 0 to 1.

The diversity of leaf endophytic bacteria can also be evaluated by hierarchical clustering of the frequencies of T-RFs in these five species (Figure 3). The frequency of a T-RF is defined as the fraction of samples of a host species that have the T-RF in question. A high frequency of a T-RF in one host species indicates that the bacterial species represented is a common component in that host species, and a low frequency means that the existence of the bacterial group represented is occasional. Complete linkage clustering of different host species based on the frequencies of T-RFs showed that *P*. *virgatum* and *S*. *nutans* were the closest to each other, and *A*. *viridis* and *R*. *humilis* were distinct from the other three species (Figure 3 (a)). These results are consistent with those obtained from the pCCA when treating host species as environmental factors. Complete linkage clustering of the T-RFs indicated different groups of the T-RFs, of which the major cluster containing the most frequent T-RFs is shown in Figure 3 (b). This cluster contains some T-RFs that are highly frequent among multiple host species. For instance, the T-RF 355 bp was highly frequent in *P*. *virgatum*, *S*. *nutans* and *A*. *psilostachya*, but rarely detected in *A*. *viridis* and *R*. *humilis*, indicating that T-RF 355 bp represents bacterial groups which are sensitive to the different physical/biochemical features of these two groups of host plant species. Some T-RFs have a high frequency in some host species but maintain a low frequency in other host species; this is interpreted to mean that the bacterial groups represented by these T-RFs are more likely to grow in the leaf endophytic bacterial communities of their preferred host species. (For complete data of the frequencies of all T-RFs, see Additional file 1: Table S5). An extreme example is the T-RF 493 bp: this T-RF had a frequency of 61.5% in *A*. *psilostachya*, but was not detected in other host species. Some unique biochemical or physiological features of *A*. *psilostachya* may lead to a preferable inner-environment for the bacterial groups represented by the T-RF 493 bp to grow, so that those bacteria are characteristic of the leaf endophytic bacterial communities in *A*. *psilostachya*.

We also calculated the average frequencies of the T-RFs over all the five host species based on the frequencies of the T-RFs in each species. The average frequency reflects the general distribution of endophytic bacteria among multiple species of host plants. In Additional file 1: Table S5, the average frequencies of all recognized T-RFs were also compared: for example, the T-RF 529 bp had an average frequency more than 80% in these five selected host species and was the most frequent T-RF.

Multivariate Analysis of Variance (MANOVA) of the T-RFLP profile also indicated that the three major factors are significant, consistent with the pCCA result. The T-RFLP profiles of all samples that include only those T-RFs present in highest proportions shown in Figure 3 (b) were also used to test the three major factors by MANOVA. Generally, for the data including all samples, Wilk’s Lambda Analysis and Hotelling-Lawley Trace Analysis both indicated that the three major factors (host species, dates and sampling sites) were significant factors at alpha = 0.05. For these nine T-RFs, at alpha = 0.05, the host species factor was significant for seven T-RFs; the sampling dates factor was significant for seven T-RFs; the sampling sites factor was significant for six T-RFs. In aggregate, these three major factors were all significant at alpha = 0.05 for four T-RFs: 75 bp, 79 bp, 236 bp and 355 bp. The three factor models for these four T-RFs gave R-square coefficients greater than 0.9. Thus, the results of MANOVA were consistent with pCCA, again confirming the importance of the three major factors.

Some prominent T-RFs were at relatively higher proportions than other T-RFs (Additional file 1: Table S5). These T-RFs represent the dominant bacterial groups in the endophytic bacterial communities. We compared APE values for the most abundant T-RFs, those which have average frequencies more than 0.3 over all five host species (Table 3 and Additional file 1: Table S6). APE values measure the relative amounts of individual T-RFs in those plants that the T-RF members have colonized. Some T-RFs were significantly different in APE among host species, making those T-RFs the characteristic T-RFs of the endophytic bacterial communities. For instance, T-RF 75 bp was much more dominant in *A*. *viridis* than it was in any of the other four species. T-RF 78 bp had an APE of 54% in *R*. *humilis* but only 7% in *S*. *nutans* and 4% in *A*. *psilostachya*; while T-RF 236 bp made up 17% of the T-RFs in *S*. *nutans*, 2% in *A*. *viridis*, but was not detected in *R*. *humilis* (Table 3). Since each T-RF represents a different group of bacteria, APE values reflect that certain groups of bacteria are present in widely different proportions in different host species, consistent with the host species determining the compositions of the endophytic bacterial communities.

**Table 3:** Average proportion per existence^a^ in five different host species of selected^b^ significant T-RFs (Average frequencies > 0.3)

**T**-**RF** (**bp**)	***A***. ***psilostachya***	***P***. ***virgatum***	***A***. ***viridis***	***S***. ***nutans***	***R***. ***humilis***
75	0.05	0.04	0.18	0.05	0.11
77	0.00	0.02	0.05	0.05	0.07
78	0.04	0.30	0.12	0.07	0.54
79	0.11	0.14	0.15	0.08	0.30
85	0.18	0.13	0.14	0.12	0.09
94	0.08	-	0.01	0.04	-
236	0.03	0.07	0.02	0.17	-
350	0.05	0.09	0.07	0.12	0.09
352	0.09	0.04	0.04	0.06	-
355	0.09	0.20	-	0.15	0.03
529	0.14	0.08	0.22	0.09	0.15

^a^ Proportions calculated for all analyzed plants of the listed plant species; “-“indicates that the T-RF was not detected in any plant of the species.

^b^ For complete listing, see Additional file 1: Table S6.

## Discussion

The Hallman et al. [8] definition of endophytic bacteria requires “surface-disinfested plant tissue” or extraction from the plant. “Disinfestation” by killing all the epiphytic bacteria may be effective when culture-dependent protocols are used, but is not appropriate in culture-independent protocols, such as the present one, since the DNA or RNA of dead epiphytes, if not removed, would still be amplified by bacteria-specific PCR. For those organs, like tubers, whose outer layers can be easily peeled off, endophytic bacteria can be isolated from inside of the plants unambiguously. However, peeling the epidermis off leaves, while possible, is not practical for a study like the present one. Therefore, to study leaf endophytic bacterial communities, it is critical to dislodge epiphytic bacteria from the leaf surfaces as far as possible. We have dislodged epiphytes using methods similar to those reported by others [13,26-28]. Since we did not test the rinse water for rDNA amplicons, we cannot be sure that we have removed all epiphytic bacteria. However, the observation that the complexities of the populations (Additional file 1: Table S5) were substantially lower than those reported for leaf epiphytic bacteria [29,30] suggests that most epiphytes have been removed.

Past studies have applied multiple enzyme digestion T-RFLP to environmental bacterial community research [31-33]. Some studies have focused on the rhizosphere, rhizoplane and the epiphytic phyllosphere bacterial communities using fingerprint techniques of 16S rRNA genes, especially the rhizosphere of single cultivated plant species including potato and rice [34-36] and the phyllosphere of soybean, rice and maize [6,37]. The present research is the first to apply single digestion T-RFLP to leaf endophytic bacteria in multiple host species. Multi-enzyme studies depend on a reliable T-RFLP database to deduce species information; however most T-RFLP databases are still developing, so that a large proportion of novel bacteria, which are highly abundant in the environment, may not be matched using current databases [21]. Although closely related bacterial species will usually produce the same T-RF, one or more other distinct taxonomic groups may also produce the same T-RF. Therefore variation in abundance of a T-RF may be due to changes in one of the represented taxonomic groups, while a second is unchanged. Multi-enzymes are used in an effort to make taxonomic assignments; however taxonomic assignments are not necessary for identification of the factorial influences on the leaf endophytic bacterial communities, as studied in this work. Single digestion T-RFLP peaks represent OTUs (Operational T-RFLP Unit) that provide information on the diversity of leaf endophytic bacteria in different environments.

In order to assess the abilities of T-RF OTUs present in individual plants to compete with other bacteria, we focused on the relative amounts of T-RF OTUs in different plants only in those plants in which they were found. The APE of a T-RF in one host species was defined as the average proportion of a T-RF in all the samples of one plant species which have this T-RF. Calculating APE rather than regular average proportion can avoid the problem of underestimation of the abundance of a T-RF in one host species due to non-infection of the bacterial species represented in some samples. The APE of a T-RF can more accurately reflect the overall compositions of leaf endophytic bacterial communities in a plant species than can methods that include absence in the analysis.

In this research, we explored the diversity of leaf endophytic bacteria in selected plant species over time and the physical environment, in order to propose a model describing how multiple factors influence endophytic bacterial communities. Past studies have found the plant genotype and growth conditions have significant impacts on the rhizosphere bacterial communities [34-36] and on the phyllosphere bacterial communities [6,38]. Here we considered three major influencing factors: host plant species, time and sampling sites. The distributions of leaf endophytic bacteria must be influenced by many factors; however, we hypothesized that these three major factors include most variables affecting community composition. We analyzed leaf endophytic bacterial communities from samples differing in these factors by pCCA and MANOVA of T-RFs and comparisons of the average amounts of T-RFs present in samples.

The factor of host plant species includes the effect of inner biochemical environment and physiological features of the host plant. The results show that the communities in the two grass species, *P*. *virgatum* and *S*. *nutans*, are similar to one another and distinct from those in the non-grass species. This may be due to similar environments inside grass plants, different from those inside the other plants. The coevolution and codivergence of host plants and leaf endophytic bacterial communities may also contribute to the similarities and differences in the leaf endophytic bacterial communities from different host species. The expectation of a major influence of host plant species on the communities was supported by distinct T-RF patterns from each host species (Figure 1 and Additional file 1: Table S5), by the results of pCCA which assigned half of the total variation to plant species, and the APE analysis (Table 3).

The time factor includes changes in the physical environment, such as temperature, humidity, irradiance and wind speed, and the dynamics of host plant growth. Jackson and Denney [27] studied the annual and seasonal variation of phyllosphere bacteria and found that compared to significant seasonal variation, the annual variation was not significant. Yadav et al. [39] also found that the mature leaves have higher populations of phyllosphere bacteria than young leaves. These studies motivated us to consider the seasonal variation of plant-associated bacteria. The pCCA examination of T-RFs treating sampling date as the environmental factor implicated it as a significant factor (Figure 2). The impacts of sampling date on the distribution of plant-associated bacteria were also seen in the average numbers of T-RFs at different sampling dates (Table 2). The temporal variations in relative abundance of different T-RFs suggest that during host plant growth, the structures of plant leaf-associated bacterial communities are also developing to respond to the changes of the inner biochemical environments of host plants and the variations of the weather and overall environment. The host species selected for study begin growth in late April or May. The ratios of the standard deviations of the average number of T-RFs to the average number are smaller in June and July than those in May and August, indicating that the plant-associated bacterial communities are more stable and complex when the host plants are growing in the peak of summer.

The factor of physical environment includes the soil and geobiochemical conditions, the effect of surrounding plants and animals, and the burning and grazing history of the sampling field, records of the latter of which are available. Again, pCCA attributed a significant contribution of sampling site to the total variation (Figure 2b) consistent with T-RF profile differences for the same plant species on the same date (Figure 1).

We recognize that the three targeted factors may not account for all the variation in the communities and that we did encounter a residual variation. Sources of this variation could include: occasional animal disturbance, insect-induced damages and other factors that cannot be measured accurately and parameterized in a mathematical model. Nevertheless, we suggest that the three-factor model describes an important part of the variation of plant-associated bacteria. The plant-associated bacterial communities are not static, but dynamic and evolve with host plants and environments.

## Conclusions

In this research of leaf endophytic bacteria, we used the method of mono-digestion T-RFLP and observed the variations of T-RFLP patterns that were contributed by three environmental factors: sampling sites, dates and host plant species. T-RFLP profiles were also analyzed by pCCA and indicated that all the three factors are statistically significant; considering the contributions to the overall variations of T-RFLP, the host plant species is the most important factor that determine the leaf endophytic bacterial communities. This discovery was also confirmed by other statistical analyses including Tukey test of the number of T-RFs, hierarchical clustering of the frequencies of T-RFs and MANOVA. These three environmental factors summarized most influencing factors and defined a well-characterized model to describe how the endophytic bacterial communities were shaped. APE was introduced to estimate the abundance of each T-RF, and dominant T-RFs have been found which represent major bacterial groups in leaf endophytic communities.

## Competing interests

The authors declare that they have no competing interests.

## Authors’ contributions

TD and UM designed the whole study and drafted the manuscript. TD and MWP designed the sampling strategy and carried out the plant sample collections. TD conducted the plant sample treatments, DNA extractions and PCR, T-RFLP and data analysis. MWP helped with data pCCA analysis and made important revisions in the manuscript. All authors read and approved the final manuscript.

## Supplementary Material

Additional file 1 **Table S1.** Locations of sampling sites in the TGPP. **Table S2**. Dominant T-RFs from amplified 16S bacterial rDNA from three plant species. **Table S3**. Summary statistics for T-RFs calculated by each host species, sampling month and sampling date **Table S5**. Frequencies of all the T-RFs in 5 different host species and their average frequencies. **Table S6**. Average Proportion per Existence (APE) of all the T-RFs in 5 different host species. Click here for file

Additional file 2 **Figure S1.** Comparison of two T-RFLP patterns of *Dde*I digestion products of the *Asclepias viridis* Sample 1 from Site 2 collected on June 16^th^, 2010, scanned on Aug 19^th^, 2010 (above) and Aug 30^th^ 2010 (below). The T-RFLP patterns of the same sample scanned in different experiments were indistinguishable, indicating that the T-RFLP is highly reproducible. Click here for file

Additional file 3 **Table S4.** T-RFLP profile Shannon alpha indeces. Click here for file
