# Supplementary material for: Community terminal restriction fragment length polymorphisms reveal insights into the diversity and dynamics of leaf endophytic bacteria
Source: BMC Microbiol. 2013 Jan 3;13:1. doi: 10.1186/1471-2180-13-1 (PMC3546043; doi:10.1186/1471-2180-13-1)
Supplement: Additional file 1 — Table S1. Locations of sampling sites in the TGPP. Table S2. Dominant T-RFs from amplified 16S bacterial rDNA from three plant species. Table S3. Summary statistics for T-RFs calculated by each host species, sampling month and sampling date Table S5. Frequencies of all the T-RFs in 5 different host species and their average frequencies. Table S6. Average Proportion per Existence (APE) of all the T-RFs in 5 different host species. [file 1471-2180-13-1-S1.doc]

**Supplementary Table 1.** Locations of sampling sites in the TGPP.

| Site No. | UTM location | Elevation (m) |
| --- | --- | --- |
| Site 1 | 14 S 0736182 4070432 | 288 |
| Site 2 | 14 S 0732625 4070095 | 300 |
| Site 3 | 14 S 0730241 4080682 | 326 |
| Site 4 | 14 S 0727969 4076948 | 299 |

**Supplementary Table 2.** Dominant T-RFs from amplified 16S bactrial rDNA from three plant species.

| Host species | Dominant T-RFs |
| --- | --- |
| *Asclepias viridis* | 75bp, 77bp, 78bp, 79bp, 85bp, 89bp, 347bp, 350bp, 354bp, 529bp * |
| *Ambrosia psilostachya* | 75bp, 79bp, 84bp, 85bp *, 94bp, 346bp, 348bp, 352bp, 355bp, 529bp |
| *Panicum virgatum* | 78bp, 79bp, 85bp, 95bp, 236bp, 355bp *, 529bp |

* indicates the most dominant T-RF in that species.

**Supplementary Table 3. Summary statistics for T-RFs calculated by each host species, sampling month and sampling date.**

| Sample Variable^a^ | Total T-RFs | Richness ^b^ | Empty cells in matrix | Beta diversity |
| --- | --- | --- | --- | --- |
| Data summarized by host species | | |  |  |
| *A. viridis* | 72 | 14.89 | 79.32% | 3.84 |
| *P. virgatum* | 60 | 15 | 75.00% | 3.00 |
| *S. nutans* | 42 | 12.92 | 69.24% | 2.25 |
| *A. psilostachya* | 52 | 17.38 | 66.59% | 1.99 |
| *R. humilis* | 16 | 5.50 | 65.63% | 1.91 |
| Data summarized by sampling date | | |  |  |
| May | 70 | 9.29 | 86.73% | 6.53 |
| June | 68 | 14.72 | 78.35% | 3.62 |
| July | 91 | 18.04 | 80.17% | 4.04 |
| August | 54 | 12.73 | 76.43% | 3.24 |
| Data summarized by site | | |  |  |
| Site 1 | 84 | 14.77 | 82.41% | 4.69 |
| Site 2 | 74 | 13.86 | 81.27% | 4.34 |
| Site 3 | 70 | 12.45 | 82.21% | 4.62 |
| Site 4 | 72 | 14.60 | 79.72% | 3.93 |

^a^ For months, data summarized over all sites; for sites, data summarized over all months.

^b^ The richness of T-RFs is defined as the average number of T-RFs in a dataset

**Supplementary Table 5.** Frequencies of all the T-RFs in 5 different host species and their average frequencies.

| T-RF (bp) | *A. psilostachya* | *P. virgatum* | *A. viridis* | *S. nutans* | *R. humilis* | Average frequency |
| --- | --- | --- | --- | --- | --- | --- |
| 55 | 0.00 | 0.00 | 0.00 | 0.00 | 0.10 | 0.02 |
| 57 | 0.08 | 0.57 | 0.00 | 0.25 | 0.00 | 0.18 |
| 62 | 0.00 | 0.07 | 0.00 | 0.00 | 0.00 | 0.01 |
| 66 | 0.00 | 0.00 | 0.03 | 0.00 | 0.00 | 0.01 |
| 67 | 0.08 | 0.00 | 0.00 | 0.00 | 0.00 | 0.02 |
| 71 | 0.08 | 0.00 | 0.17 | 0.00 | 0.00 | 0.05 |
| 72 | 0.00 | 0.21 | 0.11 | 0.17 | 0.00 | 0.10 |
| 73 | 0.00 | 0.00 | 0.25 | 0.00 | 0.00 | 0.05 |
| 74 | 0.00 | 0.14 | 0.11 | 0.00 | 0.00 | 0.05 |
| 75 | 0.85 | 0.29 | 0.75 | 0.50 | 0.40 | 0.56 |
| 76 | 0.08 | 0.14 | 0.25 | 0.08 | 0.00 | 0.11 |
| 77 | 0.00 | 0.36 | 0.75 | 0.50 | 0.50 | 0.42 |
| 78 | 0.31 | 0.57 | 0.75 | 0.58 | 0.80 | 0.60 |
| 79 | 0.85 | 0.57 | 0.19 | 0.50 | 0.40 | 0.50 |
| 81 | 0.00 | 0.00 | 0.14 | 0.00 | 0.00 | 0.03 |
| 82 | 0.00 | 0.07 | 0.00 | 0.00 | 0.00 | 0.01 |
| 83 | 0.00 | 0.00 | 0.06 | 0.00 | 0.00 | 0.01 |
| 84 | 0.23 | 0.14 | 0.00 | 0.17 | 0.00 | 0.11 |
| 85 | 0.85 | 0.71 | 0.72 | 0.67 | 0.10 | 0.61 |
| 89 | 0.00 | 0.00 | 0.92 | 0.00 | 0.00 | 0.18 |
| 92 | 0.00 | 0.00 | 0.00 | 0.17 | 0.80 | 0.19 |
| 94 | 1.00 | 0.00 | 0.06 | 0.75 | 0.00 | 0.36 |
| 95 | 0.00 | 0.93 | 0.00 | 0.00 | 0.00 | 0.19 |
| 96 | 0.00 | 0.07 | 0.11 | 0.50 | 0.10 | 0.16 |
| 97 | 0.00 | 0.00 | 0.22 | 0.00 | 0.00 | 0.04 |
| 98 | 0.00 | 0.36 | 0.00 | 0.33 | 0.10 | 0.16 |
| 99 | 0.00 | 0.00 | 0.00 | 0.08 | 0.00 | 0.02 |
| 100 | 0.00 | 0.00 | 0.00 | 0.17 | 0.00 | 0.03 |
| 103 | 0.00 | 0.00 | 0.03 | 0.00 | 0.00 | 0.01 |
| 113 | 0.00 | 0.00 | 0.03 | 0.00 | 0.00 | 0.01 |
| 129 | 0.00 | 0.07 | 0.00 | 0.00 | 0.00 | 0.01 |
| 148 | 0.00 | 0.00 | 0.03 | 0.00 | 0.00 | 0.01 |
| 163 | 0.00 | 0.00 | 0.44 | 0.00 | 0.00 | 0.09 |
| 164 | 0.31 | 0.00 | 0.14 | 0.00 | 0.00 | 0.09 |
| 185 | 0.23 | 0.00 | 0.00 | 0.00 | 0.00 | 0.05 |
| 186 | 0.08 | 0.00 | 0.00 | 0.00 | 0.00 | 0.02 |
| 193 | 0.00 | 0.00 | 0.06 | 0.00 | 0.00 | 0.01 |
| 194 | 0.00 | 0.00 | 0.03 | 0.00 | 0.00 | 0.01 |
| 203 | 0.08 | 0.00 | 0.00 | 0.00 | 0.00 | 0.02 |
| 206 | 0.00 | 0.00 | 0.03 | 0.00 | 0.00 | 0.01 |
| 213 | 0.00 | 0.00 | 0.03 | 0.00 | 0.00 | 0.01 |
| 213.8 | 0.23 | 0.00 | 0.36 | 0.00 | 0.00 | 0.12 |
| 215 | 0.08 | 0.00 | 0.25 | 0.00 | 0.00 | 0.07 |
| 219 | 0.00 | 0.00 | 0.06 | 0.00 | 0.00 | 0.01 |
| 224 | 0.00 | 0.00 | 0.03 | 0.00 | 0.00 | 0.01 |
| 224.5 | 0.31 | 0.29 | 0.03 | 0.08 | 0.00 | 0.14 |
| 227 | 0.00 | 0.00 | 0.00 | 0.08 | 0.00 | 0.02 |
| 228 | 0.54 | 0.43 | 0.03 | 0.25 | 0.00 | 0.25 |
| 229 | 0.00 | 0.00 | 0.03 | 0.00 | 0.00 | 0.01 |
| 230 | 0.00 | 0.00 | 0.03 | 0.00 | 0.00 | 0.01 |
| 232 | 0.00 | 0.21 | 0.00 | 0.00 | 0.00 | 0.04 |
| 235 | 0.31 | 0.14 | 0.42 | 0.25 | 0.00 | 0.22 |
| 236 | 0.46 | 0.50 | 0.50 | 0.33 | 0.00 | 0.36 |
| 239 | 0.08 | 0.07 | 0.08 | 0.00 | 0.00 | 0.05 |
| 241 | 0.00 | 0.07 | 0.00 | 0.08 | 0.00 | 0.03 |
| 243 | 0.15 | 0.00 | 0.00 | 0.00 | 0.00 | 0.03 |
| 245.5 | 0.00 | 0.07 | 0.00 | 0.08 | 0.00 | 0.03 |
| 247.2 | 0.00 | 0.14 | 0.00 | 0.00 | 0.00 | 0.03 |
| 248 | 0.00 | 0.07 | 0.14 | 0.17 | 0.00 | 0.08 |
| 249 | 0.08 | 0.43 | 0.03 | 0.25 | 0.00 | 0.16 |
| 251 | 0.00 | 0.07 | 0.00 | 0.00 | 0.00 | 0.01 |
| 256 | 0.00 | 0.00 | 0.00 | 0.08 | 0.00 | 0.02 |
| 266 | 0.23 | 0.14 | 0.00 | 0.08 | 0.00 | 0.09 |
| 267 | 0.00 | 0.00 | 0.03 | 0.00 | 0.00 | 0.01 |
| 268 | 0.00 | 0.07 | 0.00 | 0.08 | 0.00 | 0.03 |
| 269 | 0.08 | 0.29 | 0.00 | 0.25 | 0.00 | 0.12 |
| 280 | 0.08 | 0.00 | 0.00 | 0.00 | 0.00 | 0.02 |
| 303 | 0.00 | 0.00 | 0.03 | 0.00 | 0.00 | 0.01 |
| 319.5 | 0.00 | 0.00 | 0.03 | 0.00 | 0.00 | 0.01 |
| 327.5 | 0.08 | 0.00 | 0.00 | 0.00 | 0.00 | 0.02 |
| 335 | 0.08 | 0.00 | 0.06 | 0.00 | 0.00 | 0.03 |
| 336 | 0.00 | 0.07 | 0.00 | 0.00 | 0.00 | 0.01 |
| 337 | 0.00 | 0.00 | 0.08 | 0.00 | 0.00 | 0.02 |
| 339 | 0.00 | 0.07 | 0.00 | 0.00 | 0.00 | 0.01 |
| 345 | 0.00 | 0.00 | 0.44 | 0.00 | 0.00 | 0.09 |
| 346 | 0.77 | 0.50 | 0.00 | 0.00 | 0.10 | 0.27 |
| 347 | 0.08 | 0.14 | 0.28 | 0.17 | 0.00 | 0.13 |
| 347.5 | 0.31 | 0.00 | 0.56 | 0.00 | 0.10 | 0.19 |
| 348 | 0.69 | 0.43 | 0.00 | 0.25 | 0.00 | 0.27 |
| 349 | 0.00 | 0.07 | 0.00 | 0.58 | 0.20 | 0.17 |
| 350 | 0.62 | 0.79 | 0.69 | 0.58 | 0.70 | 0.68 |
| 351 | 0.31 | 0.14 | 0.06 | 0.00 | 0.00 | 0.10 |
| 352 | 0.62 | 0.43 | 0.56 | 0.58 | 0.00 | 0.44 |
| 353 | 0.00 | 0.07 | 0.03 | 0.42 | 0.10 | 0.12 |
| 354 | 0.00 | 0.00 | 0.94 | 0.00 | 0.00 | 0.19 |
| 355 | 1.00 | 1.00 | 0.00 | 1.00 | 0.30 | 0.66 |
| 367 | 0.00 | 0.00 | 0.14 | 0.00 | 0.00 | 0.03 |
| 368 | 0.77 | 0.43 | 0.00 | 0.25 | 0.00 | 0.29 |
| 370 | 0.00 | 0.00 | 0.03 | 0.00 | 0.00 | 0.01 |
| 372 | 0.00 | 0.14 | 0.00 | 0.00 | 0.00 | 0.03 |
| 375 | 0.00 | 0.14 | 0.00 | 0.00 | 0.00 | 0.03 |
| 376 | 0.00 | 0.00 | 0.03 | 0.00 | 0.00 | 0.01 |
| 377 | 0.15 | 0.07 | 0.00 | 0.00 | 0.00 | 0.05 |
| 379 | 0.08 | 0.07 | 0.00 | 0.00 | 0.00 | 0.03 |
| 380 | 0.00 | 0.07 | 0.31 | 0.00 | 0.00 | 0.08 |
| 380.7 | 0.69 | 0.36 | 0.25 | 0.42 | 0.00 | 0.34 |
| 382 | 0.00 | 0.07 | 0.03 | 0.00 | 0.00 | 0.02 |
| 383 | 0.15 | 0.00 | 0.00 | 0.00 | 0.00 | 0.03 |
| 384 | 0.15 | 0.00 | 0.00 | 0.00 | 0.00 | 0.03 |
| 395 | 0.00 | 0.00 | 0.03 | 0.00 | 0.00 | 0.01 |
| 396 | 0.08 | 0.00 | 0.00 | 0.00 | 0.00 | 0.02 |
| 397 | 0.00 | 0.07 | 0.00 | 0.00 | 0.00 | 0.01 |
| 493 | 0.62 | 0.00 | 0.00 | 0.00 | 0.00 | 0.12 |
| 504 | 0.00 | 0.00 | 0.03 | 0.00 | 0.00 | 0.01 |
| 509 | 0.08 | 0.00 | 0.00 | 0.08 | 0.00 | 0.03 |
| 511 | 0.00 | 0.07 | 0.03 | 0.00 | 0.00 | 0.02 |
| 513 | 0.00 | 0.00 | 0.03 | 0.00 | 0.00 | 0.01 |
| 514 | 0.00 | 0.00 | 0.06 | 0.00 | 0.00 | 0.01 |
| 523 | 0.08 | 0.07 | 0.06 | 0.08 | 0.00 | 0.06 |
| 524 | 0.23 | 0.14 | 0.19 | 0.00 | 0.00 | 0.11 |
| 525 | 0.23 | 0.07 | 0.53 | 0.00 | 0.00 | 0.17 |
| 526 | 0.54 | 0.07 | 0.08 | 0.00 | 0.00 | 0.14 |
| 527 | 0.15 | 0.14 | 0.00 | 0.17 | 0.00 | 0.09 |
| 528 | 0.00 | 0.07 | 0.00 | 0.00 | 0.00 | 0.01 |
| 529 | 1.00 | 0.79 | 0.83 | 0.75 | 0.70 | 0.81 |
| 531 | 0.00 | 0.00 | 0.03 | 0.00 | 0.00 | 0.01 |
| 532 | 0.08 | 0.14 | 0.00 | 0.00 | 0.00 | 0.04 |
| 536 | 0.00 | 0.00 | 0.03 | 0.00 | 0.00 | 0.01 |
| 550 | 0.08 | 0.00 | 0.00 | 0.00 | 0.00 | 0.02 |
| 624 | 0.00 | 0.00 | 0.00 | 0.08 | 0.00 | 0.02 |
| 672 | 0.00 | 0.00 | 0.03 | 0.00 | 0.00 | 0.01 |
| 706 | 0.00 | 0.00 | 0.03 | 0.00 | 0.00 | 0.01 |

**Supplementary Table 6.** Average Proportion per Existence (APE) of all the T-RFs in 5 different host species.

| T-RF (bp) | *A. psilostachya* | *P. virgatum* | *A. viridis* | *S. nutans* | *R. humilis* |
| --- | --- | --- | --- | --- | --- |
| 55 | - | - | - | - | 0.01 |
| 57 | 0.02 | 0.05 | - | 0.03 | - |
| 62 | - | 0.00 | - | - | - |
| 66 | - | - | 0.00 | - | - |
| 67 | 0.00 | - | - | - | - |
| 71 | 0.02 | - | 0.01 | - | - |
| 72 | - | 0.01 | 0.01 | 0.03 | - |
| 73 | - | - | 0.01 | - | - |
| 74 | - | 0.02 | 0.01 | - | - |
| 75 | 0.05 | 0.04 | 0.18 | 0.05 | 0.11 |
| 76 | 0.00 | 0.01 | 0.02 | 0.03 | - |
| 77 | - | 0.02 | 0.05 | 0.05 | 0.07 |
| 78 | 0.04 | 0.30 | 0.12 | 0.07 | 0.54 |
| 79 | 0.11 | 0.14 | 0.15 | 0.08 | 0.30 |
| 81 | - | - | 0.03 | - | - |
| 82 | - | 0.00 | - | - | - |
| 83 | - | - | 0.01 | - | - |
| 84 | 0.01 | 0.04 | - | 0.02 | - |
| 85 | 0.18 | 0.13 | 0.14 | 0.12 | 0.09 |
| 89 | - | - | 0.09 | - | - |
| 92 | - | - | - | 0.09 | 0.11 |
| 94 | 0.08 | - | 0.01 | 0.04 | - |
| 95 | - | 0.07 | - | - | - |
| 96 | - | 0.11 | 0.04 | 0.09 | 0.10 |
| 97 | - | - | 0.02 | - | - |
| 98 | - | 0.05 | - | 0.09 | 0.02 |
| 99 | - | - | - | 0.02 | - |
| 100 | - | - | - | 0.32 | - |
| 103 | - | - | 0.07 | - | - |
| 113 | - | - | 0.01 | - | - |
| 129 | - | 0.01 | - | - | - |
| 148 | - | - | 0.05 | - | - |
| 163 | - | - | 0.03 | - | - |
| 164 | 0.01 | - | 0.03 | - | - |
| 185 | 0.03 | - | - | - | - |
| 186 | 0.03 | - | - | - | - |
| 193 | - | - | 0.03 | - | - |
| 194 | - | - | 0.04 | - | - |
| 203 | 0.00 | - | - | - | - |
| 206 | - | - | 0.00 | - | - |
| 213 | - | - | 0.01 | - | - |
| 213.8 | 0.02 | - | 0.01 | - | - |
| 215 | 0.05 | - | 0.01 | - | - |
| 219 | - | - | 0.01 | - | - |
| 224 | - | - | 0.01 | - | - |
| 224.5 | 0.02 | 0.01 | 0.01 | 0.02 | - |
| 227 | - | - | - | 0.03 | - |
| 228 | 0.02 | 0.04 | 0.02 | 0.13 | - |
| 229 | - | - | 0.01 | - | 0.00 |
| 230 | - | - | 0.01 | - | - |
| 232 | - | 0.01 | - | - | - |
| 235 | 0.02 | 0.02 | 0.02 | 0.01 | - |
| 236 | 0.03 | 0.07 | 0.02 | 0.17 | - |
| 239 | 0.01 | 0.01 | 0.00 | - | - |
| 241 | - | 0.02 | - | 0.02 | - |
| 243 | 0.01 | - | - | - | - |
| 245.5 | - | 0.00 | - | 0.01 | - |
| 247.2 | - | 0.01 | - | - | - |
| 248 | - | 0.01 | 0.01 | 0.02 | - |
| 249 | 0.01 | 0.03 | 0.01 | 0.17 | - |
| 251 | - | 0.00 | - | - | - |
| 256 | - | - | - | 0.02 | - |
| 266 | 0.02 | 0.01 | - | 0.01 | - |
| 267 | - | - | 0.01 | - | - |
| 268 | - | 0.00 | - | 0.03 | - |
| 269 | 0.01 | 0.03 | - | 0.03 | - |
| 280 | 0.01 | - | - | - | - |
| 303 | - | - | 0.04 | - | - |
| 319.5 | - | - | 0.00 | - | - |
| 327.5 | 0.03 | - | - | - | - |
| 335 | 0.00 | - | 0.02 | - | - |
| 336 | - | 0.00 | - | - | - |
| 337 | - | - | 0.01 | - | - |
| 339 | - | 0.00 | - | - | - |
| 345 | - | - | 0.02 | - | - |
| 346 | 0.06 | 0.01 | - | - | 0.03 |
| 347 | 0.00 | 0.00 | 0.05 | 0.01 | - |
| 347.5 | 0.03 | - | 0.06 | - | 0.18 |
| 348 | 0.09 | 0.02 | - | 0.06 | - |
| 349 | - | 0.00 | - | 0.07 | 0.25 |
| 350 | 0.05 | 0.09 | 0.07 | 0.12 | 0.09 |
| 351 | 0.04 | 0.01 | 0.01 | - | - |
| 352 | 0.09 | 0.04 | 0.04 | 0.06 | - |
| 353 | - | 0.39 | 0.00 | 0.03 | 0.12 |
| 354 | - | - | 0.09 | - | - |
| 355 | 0.09 | 0.20 | - | 0.15 | 0.03 |
| 367 | - | - | 0.01 | - | - |
| 368 | 0.02 | 0.03 | - | 0.07 | - |
| 370 | - | - | 0.00 | - | - |
| 372 | - | 0.02 | - | - | - |
| 375 | - | 0.00 | - | - | - |
| 376 | - | - | 0.01 | - | - |
| 377 | 0.01 | 0.00 | - | - | - |
| 379 | 0.01 | 0.00 | - | - | - |
| 380 | - | 0.01 | 0.02 | - | - |
| 380.7 | 0.02 | 0.01 | 0.01 | 0.02 | - |
| 382 | - | 0.02 | 0.01 | - | - |
| 383 | 0.02 | - | - | - | - |
| 384 | 0.01 | - | - | - | - |
| 395 | - | - | 0.02 | - | - |
| 396 | 0.02 | - | - | - | - |
| 397 | - | 0.00 | - | - | - |
| 493 | 0.03 | - | - | - | - |
| 504 | - | - | 0.01 | - | - |
| 509 | 0.01 | - | - | 0.03 | - |
| 511 | - | 0.00 | 0.01 | - | - |
| 513 | - | - | 0.01 | - | - |
| 514 | - | - | 0.01 | - | - |
| 523 | 0.01 | 0.00 | 0.01 | 0.01 | - |
| 524 | 0.02 | 0.01 | 0.02 | - | - |
| 525 | 0.03 | 0.01 | 0.02 | - | - |
| 526 | 0.01 | - | 0.01 | - | - |
| 527 | 0.02 | 0.02 | - | 0.01 | - |
| 528 | - | 0.00 | - | - | - |
| 529 | 0.14 | 0.08 | 0.22 | 0.09 | 0.15 |
| 531 | - | - | 0.01 | - | - |
| 532 | 0.01 | 0.01 | - | - | - |
| 536 | - | - | 0.01 | - | - |
| 550 | 0.03 | - | - | - | - |
| 624 | - | - | - | 0.02 | - |
| 672 | - | - | 0.02 | - | - |
| 706 | - | - | 0.00 | - | - |

“-“ indicates that the T-RF was not detected in any plant of the species.
