# Supplementary figures and images for: Community terminal restriction fragment length polymorphisms reveal insights into the diversity and dynamics of leaf endophytic bacteria
Source: BMC Microbiol. 2013 Jan 3;13:1. doi: 10.1186/1471-2180-13-1 (PMC3546043; doi:10.1186/1471-2180-13-1)

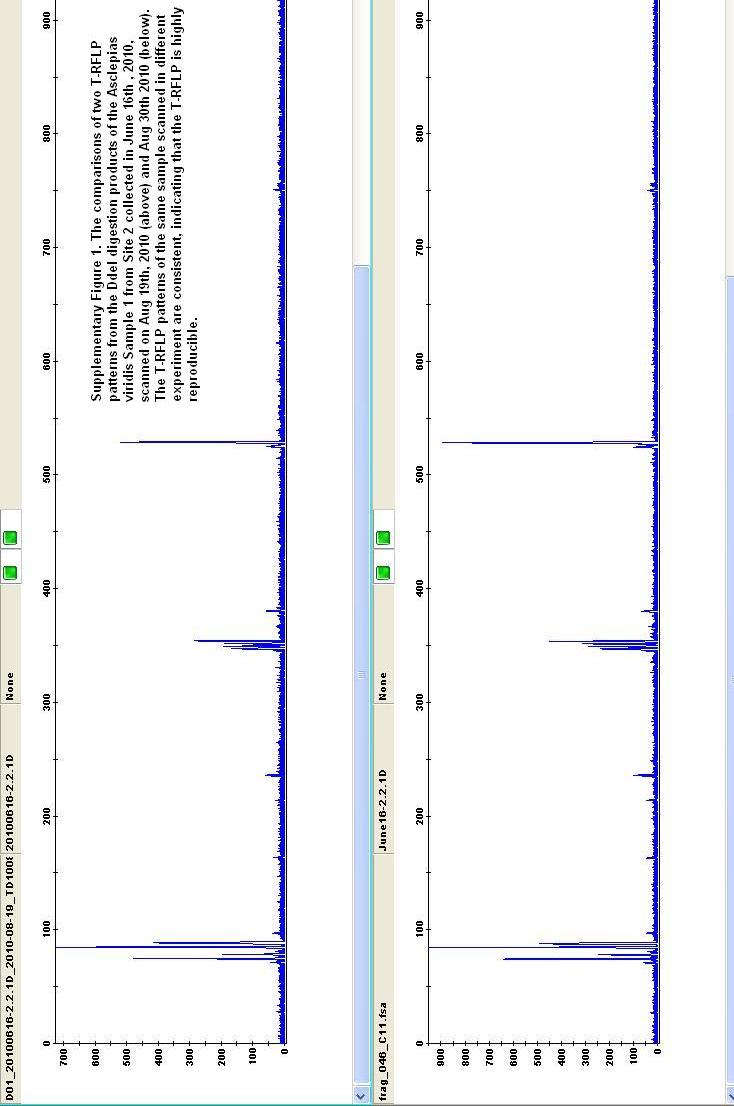

Supplement: Additional file 2 — Figure S1. Comparison of two T-RFLP patterns of DdeI digestion products of the Asclepias viridis Sample 1 from Site 2 collected on June 16th, 2010, scanned on Aug 19th, 2010 (above) and Aug 30th 2010 (below). The T-RFLP patterns of the same sample scanned in different experiments were indistinguishable, indicating that the T-RFLP is highly reproducible. [file 1471-2180-13-1-S2.jpeg]
